# Microbial community dynamics in mesophilic and thermophilic batch reactors under methanogenic, phenyl acid-forming conditions

**DOI:** 10.1186/s13068-020-01721-z

**Published:** 2020-05-06

**Authors:** Eva Maria Prem, Blaz Stres, Paul Illmer, Andreas Otto Wagner

**Affiliations:** 1 Department of Microbiology, Universität Innsbruck, Technikerstraße 25d, 6020 Innsbruck, Austria; 2 Department of Animal Science, Biotechnical Faculty, University of Ljubljana, Jamnikarjeva 101, 1000 Ljubljana, Slovenia; 3 Institute of Sanitary Engineering, Faculty of Civil and Geodetic Engineering, University of Ljubljana, Jamova 2, 1000 Ljubljana, Slovenia; 4 Department of Automation, Biocybernetics and Robotics, Jozef Štefan Institute, Jamova 39, 1000 Ljubljana, Slovenia

**Keywords:** Anaerobic digestion, Phenylacetate, Phenylpropionate, Aromatic compounds, Biogas, *Next*-*generation* sequencing, *Piphillin* analyses

## Abstract

**Background:**

Proteinaceous wastes exhibit high theoretical methane yields and their residues are considered valuable fertilisers. The routine anaerobic degradation of proteins often raises problems like high aromatic compound concentrations caused by the entry of aromatic amino acids into the system. A profound investigation of the consequences of aromatic compound exposure on various microorganisms, which cascade-like and interdependently degrade complex molecules to biogas, is still pending.

**Results:**

In mesophilic samples, methane was predominantly produced via acetoclastic methanogenesis. The highest positive correlation was observed between phenylacetate (PAA) and *Psychrobacter* spp. and between phenylpropionate (PPA) and *Haloimpatiens* spp. Moreover, *Syntrophus* spp. negatively correlated with PAA (Spearman’s rank correlations coefficient (rs) = − 0.46, *p* < 0.05) and PPA concentrations (rs = − 0.44, *p* < 0.05) and was also associated with anaerobic benzene ring cleavage. In thermophilic samples, acetate was predominantly oxidised by *Tepidanaerobacter* spp. or *Syntrophaceticus* spp. in syntrophic association with a hydrogenotrophic methanogen. The genera *Sedimentibacter* and *Syntrophaceticus* correlated positively with both PAA and PPA concentrations. Moreover, *Sedimentibacter* spp., *Tepidanaerobacter* spp., *Acetomicrobium* spp., and *Sporanaerobacter* spp. were significant *LEfSe* (linear discriminant analysis effect size) biomarkers for high meso- as well as thermophilic phenyl acid concentrations. Direct negative effects of phenyl acids on methanogenic properties could not be proven.

**Conclusions:**

Anaerobic phenyl acid formation is not restricted to specific microbial taxa, but rather done by various meso- and thermophilic bacteria. The cleavage of the highly inert benzene ring is possible in methanogenic batch reactors—at least in mesophilic fermentation processes. The results indicated that phenyl acids rather affect microorganisms engaged in preceding degradation steps than the ones involved in methanogenesis.

## Background

On a global scale, waste products coming from food industry or from agriculture are available in large quantities. Using waste products of the respective region for biogas formation can be an economically effective and sustainable way to contribute to the renewable energy pool [1, 2]. Biogas reactors rely on cascade-like interactions between various microorganisms that interdependently degrade complex substrates to methane and carbon dioxide [3]. However, an increased use of waste products can be challenging due to undesirable compounds entering biogas plants [4–7]. Protein-rich waste products like slaughterhouse waste, thin stillage, or pig manure have indeed a high theoretical methane yield [8–10] and the resulting residues are considered desirable fertilisers [11]. However, the anaerobic degradation of proteins is often problematic due to the rise of ammonia [8, 11, 12] or hydrogen sulphide [10]. Free ammonia is particularly toxic to acetoclastic methanogens; therefore, syntrophic acetate oxidation (SAO) combined with hydrogenotrophic methanogenesis is a common pathway in ammonia-rich anaerobic reactors [8–10].

Aromatic compounds are another group of potentially problematic materials [7, 13–16]. They are one of the most abundant organic compounds on earth and enter the biogas reactors via proteins, lignocellulosic materials, and pollutants [17]. Tryptophan (Tryp), tyrosine (Tyr), and phenylalanine (Phe) are aromatic amino acids thus contain a benzene ring, which is very stable due to its six-carbon-joined planar ring structure [16]. They enter the biogas reactor via proteins, depending on the respective composition of the substrate [18].

Despite the ubiquitous occurrence of aromatic compounds, only microorganisms (prokaryotes and fungi) are able to completely degrade these materials [19]. Since the 1980s, several studies showed that not only aerobic, but also anaerobic benzene degradation is possible under certain electron accepting conditions (for example under methanogenic or sulphate-reducing conditions) [20–22]. The anaerobic degradation of aromatic compounds—albeit considered distinctly slower than the aerobic approach—plays an important role in biogeochemical cycles as aromatic compounds are present in abundance in various anoxic habitats [23, 24]. The phenyl acids phenylacetate (PAA) and phenylpropionate (PPA), two monocyclic aromatic acids, are relevant aromatic intermediates in the anaerobic degradation of benzenes [4, 7, 25]; however, these two compounds received little attention so far [15]. Anaerobic Tyr and Phe degradation by fermenting bacteria was shown to lead to the formation of PAA and 4-hydroxyphenylacetate, respectively [23]. Some *Clostridia* were shown to degrade Phe to phenyllactate (PLA) and subsequently to PAA without attacking the benzene ring itself [23, 26]. One key enzyme in the Phe degradation is the phenylacetaldehyde dehydrogenase responsible for the conversion of phenylacetaldehyde to PAA as shown with the model organisms *Aromatoleum aromaticum* and *Thauera aromatica* [27, 28].

Depending on the respective substituents, aromatic compounds are further anaerobically degraded via special central intermediates [19]. PAA and PPA are degraded to the intermediate benzoyl-CoA [23]. Once formed, benzoyl-CoA enters the central pathway leading to the de-aromatisation and (hydrolytic) cleavage of the phenyl ring [14, 23, 29–31]; albeit facultative and obligate anaerobic microorganisms use different enzymes during the benzoyl-CoA reduction [29]. In *Thauera aromatica*, benzoyl-CoA is further reduced to cyclohexa-1,5-diene-1-carbonyl-CoA by a benzoyl-CoA reductase. The next steps include a hydratase and a dehydrogenase. Ring cleavage finally takes place by adding H_2_O to the double bound of 6-oxocyclohex-1-ene-1-carbonyl-CoA by 6-oxocyclohex-1-ene-1-carbonyl-CoA hydrolase (Kyoto Encyclopedia of Genes and Genomes (KEGG) orthology K07539), which results in the formation of 3-hydroxypimelyl-CoA [29]. Anaerobic benzoate degradation via benzoyl-CoA has also been profoundly studied in model organisms other than *Thauera aromatica*, like *Azoarcus* spp. or *Geobacter metallireducens* [23, 28, 32–36]. Some model organisms are able to carry out several degradation steps within the respective peripheral and/or central pathway [27, 33]. Under more natural conditions, due to the complex microbial interactions and interdependencies, it is more likely that a variety of microbial species take part in the degradation of aromatic compounds [31]. By contrast, tryptophan is characterised by an indole ring system and is anaerobically degraded to 2-aminobenzoyl-CoA using enzymes like 2-aminobenzoate-CoA ligase [23].

The effects of aromatic compounds on microorganisms in methanogenic communities are still not clear due to the previous use of different aromatic compounds, temperature regimes, and inocula. For instance, a single PAA pulse was shown to be responsible for an archaeal shift from acetoclastic to hydrogenotrophic methanogens in primary sludge digesters at mesophilic temperatures, whereas the archaeal communities were more stable in digesters containing primary/secondary sludge mixtures [4]. Moreover, PAA concentrations above 0.5 g L^−1^ led to clear inhibitory responses in thermophilic bioreactors [37]. By contrast, PAA and PPA were shown to have a stimulatory effect on the cellulose-degrader *Ruminococcus albus* [38, 39].

Wagner et al. [15] simulated different stages of overload using mesophilic and thermophilic batch communities and evaluated phenyl acid generation (PAA and PPA) and biogas production performance. Phenyl acid formation could be observed at certain overload conditions. PAA and PPA did not necessarily lead to a low methane generation [5, 15]. Substrate load rather than temperature or inoculum was shown to influence PAA and PPA turnover [15]. In the present study, samples derived from this data set [15] were subjected to microbiological analysis in order (i) to give an overview of microbial shifts during anaerobic digestion (AD) of amino acids and proteinaceous substrates under different overload and temperature conditions; (ii) to investigate microorganisms involved in methanogenesis in more detail; (iii) to correlate the formation and degradation of phenyl acids to specific genera/microbial groups, and (iv) to search for general peripheral as well as central benzoyl-CoA pathways and for microbial enzymes associated with anaerobic cleavage of the benzene ring.

## Results

### Mesophilic and thermophilic community composition

Prior to filtering, 1661 operational taxonomic units (OTUs) were generated in total. Consequently, and irrespective of the overload conditions, the microbial diversity (Shannon Index) was considerably higher in mesophilic than in thermophilic samples as shown in Additional file 1: Fig. S3. Therefore, data were thenceforward analysed separately. To remove noisy OTU categories, OTUs with a total abundance below 10 were excluded from each temperature regime (abundance per sample of removed OTUs: ≤ 5). Thereafter, 659 OTUs and 282 OTUs remained for further analyses in mesophilic and thermophilic samples, respectively.

In total, 38 bacterial and five archaeal phyla were found in mesophilic samples. The most abundant mesophilic phyla were *Bacteroidetes*, *Firmicutes,* and *Chloroflexi*. The most abundant phylum in Tryp, Tyr, and control (Cont) samples was *Bacteroidetes* with a mean sequence abundance of 28% in Tryp and 29% in both Tyr and Cont samples. In Cas and ME samples, *Firmicutes* was the dominant phylum with a mean sequence abundance of 39% (Cas) and 42% (ME). In Phe samples, the contribution of *Bacteroidetes* and *Firmicutes* was balanced (25% *Firmicutes* and 24% *Bacteroidetes*). The relative sequence abundance of the phylum *Chloroflexi* was highest in the high PAA concentration group (low: 14%, medium: 12%, and high: 22%). By contrast, the abundance of *Bacteroidetes* was lower at higher PAA concentrations (low: 26%, medium: 23%, and high: 19%). The phylum *Firmicutes* dominated at high PPA concentrations (low: 26%, medium: 45%, and high: 55%). Significant phyla with an effect size ≥ 1 are depicted in Additional file 1: Fig. S1 for low and high PAA and PPA concentrations. A comprehensive overview of mesophilic communities can be looked up in the respective KRONA file (Additional file 1: Fig. S4).

In contrast to mesophilic samples, only 19 bacterial and two archaeal phyla were associated with thermophilic samples. The phyla *Thermotogae* and *Firmicutes* dominated the thermophilic communities. The mean relative sequence abundance of *Thermotogae* (all sequences of this phylum were classified as genus *Defluviitoga*) was especially high in amino acid samples (Tryp: 61%, Tyr: 54%, and Phe: 60%). The abundance of the phylum *Firmicutes* was highest in complex protein samples (ME: 57% and Cas: 56%). In the high PAA concentration group, the phylum *Firmicutes* was prevailing (relative abundance: 55%), whereas *Thermotogae* was dominant in the medium PAA concentration group (55%). The abundance of the phylum *Synergistetes* was relatively high at elevated PAA and PPA levels. Phyla with an effect size ≥ 1 for low and high PAA and PPA concentration are depicted in Additional file 1: Fig. S2. A comprehensive overview of thermophilic communities can be found in the respective KRONA file (Additional file 1: Fig. S5).

### Mesophilic communities

#### Core microbiome and metagenomic biomarkers

Core members for each substrate are listed in Table 1. The genera ADurb.Bin120 (*Anaerolineaceae*), *Anaerolineaceae* (uncultured genus), Bacteroidetes_vadinHA17_genus, and *Fastidiosipila* were part of each mesophilic core microbiome, irrespective of the substrate or variation. The acetoclastic methanogen *Methanosaeta* was a core member of the control and Phe samples; no other methanogen could be detected in any mesophilic core microbiome.

**Table 1:** List of genera defining the core microbiome of each substrate over all time points

Substrate	Mesophilic		Thermophilic	
	Sample size	Core microbiome	Sample size	Core microbiome
Cont	9	ADurb.Bin120 (*Anaerolineaceae*)^a^ *Anaerolineaceae* (uncultured)^a^ *Macellibacteroides* *Proteiniphilum* *Bacteroidetes*_vadinHA17^a^ Candidatus *Cloacimonas* *Fastidiosipila*^a^ *Methanosaeta* *Synergistaceae* (uncultured) *Cloacimonadaceae*_W5	8	*Defluviitoga*^b^ *Caldicoprobacter* DTU014 (*Clostridia*)^b^ MBA03 (*Clostridia*) *Firmicutes* (uncultured)
Tryp	12	ADurb.Bin120 (*Anaerolineaceae*)^a^ *Anaerolineaceae* (uncultured)^a^ *Macellibacteroides* *Proteiniphilum* *Bacteroidetes*_vadinHA17^a^ *Fastidiosipila*^a^ *Synergistaceae (*uncultured_genus 1)	12	*Defluviitoga*^b^ *Caldicoprobacter* DTU014 (*Clostridia*)^b^ MBA03 (*Clostridia*) *Syntrophaceticus*
Tyr	12	ADurb.Bin120 (*Anaerolineaceae*)^a^ *Anaerolineaceae* (uncultured)^a^ *Macellibacteroides* *Proteiniphilum* *Bacteroidetes*_vadinHA17^a^ *Fastidiosipila*^a^ *Christensenellaceae*_R-7_group *Synergistaceae* (uncultured_genus 1)	12	*Defluviitoga*^b^ *Caldicoprobacter* DTU014 (*Clostridia*)^b^ *Ruminiclostridium* *Syntrophaceticus*
Phe	12	ADurb.Bin120 (*Anaerolineaceae*)^a^ *Anaerolineaceae* (uncultured)^a^ *Proteiniphilum* *Bacteroidetes*_vadinHA17^a^ Candidatus *Cloacimonas* *Fastidiosipila*^a^ *Methanosaeta*	12	*Defluviitoga*^b^ DTU014 (*Clostridia*)^b^ *Syntrophaceticus*
ME	18	ADurb.Bin120 (*Anaerolineaceae*)^a^ *Anaerolineaceae* (uncultured)^a^ *Bacteroidetes*_vadinHA17^a^ Candidatus *Cloacimonas* *Fastidiosipila*^a^	17	*Defluviitoga*^b^ *Caldicoprobacter* DTU014 (*Clostridia*)^b^ *Proteiniphilum* *Tepidanaerobacter* *Sporanaerobacter* MBA03 (*Clostridia*)
Cas	18	ADurb.Bin120 (*Anaerolineaceae*)^a^ *Anaerolineaceae* (uncultured)^a^ *Proteiniphilum* *Bacteroidetes*_vadinHA17^a^ Candidatus *Cloacimonas* *Fastidiosipila*^a^ *Sedimentibacter* *Ruminococcaceae* (uncultured)	18	*Defluviitoga*^b^ *Caldicoprobacter* DTU014 (*Clostridia*)^b^ *Tepidanaerobacter* MBA03 (*Clostridia*) *Gelria*

Genera marked with ^a^ and ^b^ were part of every mesophilic and thermophilic core microbiome, respectively

Significant biomarkers with a linear discriminant analysis (LDA) score ≥ 4 are listed in Table 2 for all substrates. The Cont, Tryp, and Tyr samples showed considerably more metagenomic biomarkers than Phe, ME, and Cas samples. Via the *LEfSe* (linear discriminant analysis effect size) algorithm using the substrate as class and the degree of overload (low, medium, high) as subclass, *Methanoculleus* spp. was shown to be a significant biomarker for Cas samples. Only few mesophilic microorganisms were core members as well as significant biomarkers: *Methanosaeta* and Candidatus_Cloacimonas for the controls, *Proteiniphilum* for Tryp samples, and *Christensenellaceae*_R-7_group for Tyr samples (Tables 1 and 2).

**Table 2:** Significant *LEfSe* biomarkers with a linear discriminant analysis (LDA) score ≥ 4, using the respective substrate as class and the degree of overload (low, medium, high) as subclass

Substrate (class)	Mesophilic		Thermophilic	
	Sample size	*LEfSe* biomarkers	Sample size	*LEfSe* biomarkers
Cont	9	Candidatus *Cloacimonas* *Methanosaeta* *Pedosphaeraceae*_genus *Gracilibacter*	8	*Lachnospiraceae* (uncultured genus) *Halocella*
Tryp	6	*Proteiniphilum* *Desulfitobacterium*	6	–
Tyr	6	*Christensenellaceae*_R-7_group *Lachnoclostridium*_5 *Treponema*_2 *Methanobacterium*	6	*Tepidimicrobium*
Phe	6	–	6	–
ME	6	*Paraclostridium*	5	*Sporanaerobacter* *Proteiniphilum*
Cas	6	*Romboutsia* *Methanoculleus*	6	*Gelria* *Tepidanaerobacter*

Sample sizes refer to each substrate–overload combination over all time measuring points for each temperature regime

#### Phenyl acids and community dynamics

Results of mesophilic phenyl acid formation were published previously [15] and are depicted in a summarised form in Additional file 1: Fig. S6. During mesophilic incubation, the controls did not form any phenyl acids, whereas all reactors containing additional substrates showed high phenyl acid concentrations. After 28 days, the highest PAA concentrations were found in Phe samples under medium load conditions; the highest PPA concentrations were detected in casein-fed reactors under high load conditions [15].

**Fig. 1:**
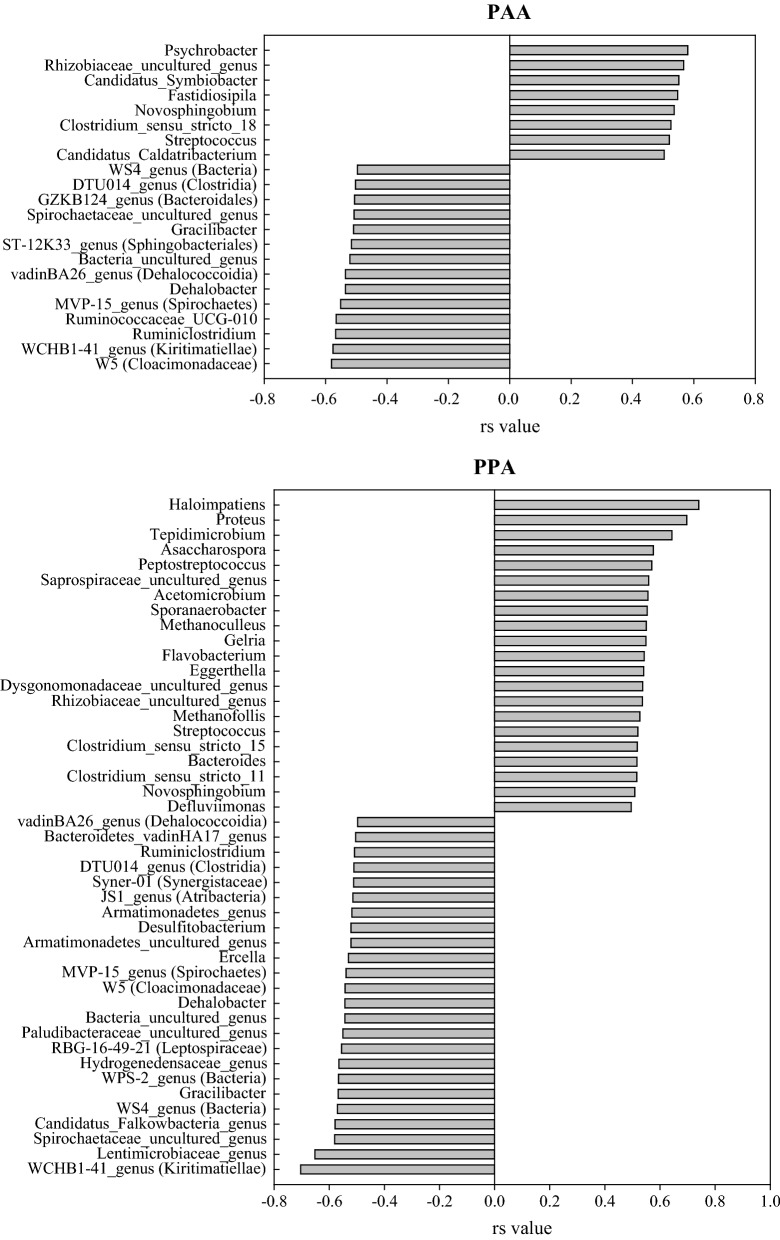
*Spearman*’s rank correlation coefficients (rs) including B–H adjustments between mesophilic genera and PAA and between mesophilic genera and PPA concentrations of day 28; Visualisation is restricted to genera with rs values ≤ − 0.50 or ≥ 0.50. OTU’s with a standard deviation < 3 calculated over all samples were excluded

*Spearman* correlations (*Benjamini*–*Hochberg* (B–H) adjusted) were calculated for samples of day 28. More meso- than thermophilic taxa correlated (*p* < 0.05) with phenyl acid concentrations. *Spearman* rank correlation coefficients were also higher in meso- than in thermophilic samples. The highest positive (*p* < 0.05) correlations between PAA concentration and microbial genera could be shown with *Psychrobacter, Rhizobiaceae* (uncultured genus), and Candidatus_Symbiobacter (Fig. 1). Furthermore, PAA concentration was negatively (*p* < 0.05) correlated with several genera including W5 (*Cloacimonadaceae*), WCHB1-41 (*Kiritimatiellae*), and *Ruminiclostridium* (Fig. 1).

PPA concentration correlated highly positive (*p* < 0.05) with several mesophilic genera including *Haloimpatiens, Proteus,* and *Tepidimicrobium* (Fig. 1). Negative *Spearman* correlations (*p* < 0.05) were observed between PPA concentrations and genera like WCHB1-41 (*Kiritimatiellae*) or *Lentimicrobiaceae* (uncultured genus) (Fig. 1).

The *LEfSe* algorithm was used to search for significant biomarkers. Significant biomarkers with an LDA score of ≥ 4 for high PAA samples were genera like *Sedimentibacter,* ADurb.Bin120 (*Anaerolineaceae*), or *Anaerolineaceae* (uncultured genus). Significant biomarkers with an LDA score ≥ 4 for the high PPA concentration group included genera like *Tepidanaerobacter, Syntrophomonas*, or *Anaerosalibacter*. A detailed list of significant biomarkers for the high PAA and PPA concentration groups can be found in Table 3. For mesophilic *LEfSe* biomarkers of low and medium PAA and PPA concentration groups, please refer to Additional file 1: Table S1.

**Table 3:** Meso- (upper row) and thermophilic (lower row) *LEfSe* biomarker with a LDA score ≥ 4 of the respective high PAA (left column) or high PPA concentration group (right column)

	PAA (class)	Sample size	*LEfSe* biomarkers	PPA (class)	Sample size	*LEfSe* biomarkers
Mesophilic	High	12	***Sedimentibacter*** ADurb.Bin120 (*Anaerolineaceae)* *Anaerolineaceae* (uncultured genus) *Tyzzerella* *Fastidiosipila* *Caproiciproducens* *Bacteroidetes*_vadinHA17_genus Candidatus *Caldatribacterium* *Christensenellaceae*_R-7_group *Ruminococcaceae*_genus	High	5	***Tepidanaerobacter*** *Syntrophomonas* MBA03_genus (*Clostridia*) *Anaerosalibacter* *Firmicutes* (uncultured genus) *Terrisporobacter* *Proteiniborus* *Methanoculleus* *Tepidimicrobium* ***Sporanaerobacter*** *Clostridiales*_FamilyXI (uncultured genus) SRB2_genus (*Clostridia*) ***Acetomicrobium*** *Aminobacterium* *Ruminococcaceae* (uncultured genus) *Fermentimonas*
Thermophilic	High	9	*Keratinibaculum* DTU014_genus (*Clostridia*) ***Tepidanaerobacter*** ***Acetomicrobium*** *Lactobacillus*	High	15	***Sporanaerobacter*** ***Acetomicrobium*** *Clostridium*_sensu_stricto_18 ***Sedimentibacter***

Genera in bold are biomarkers in meso- as well as thermophilic samples

#### Methanogenic properties

**Fig. 2:**
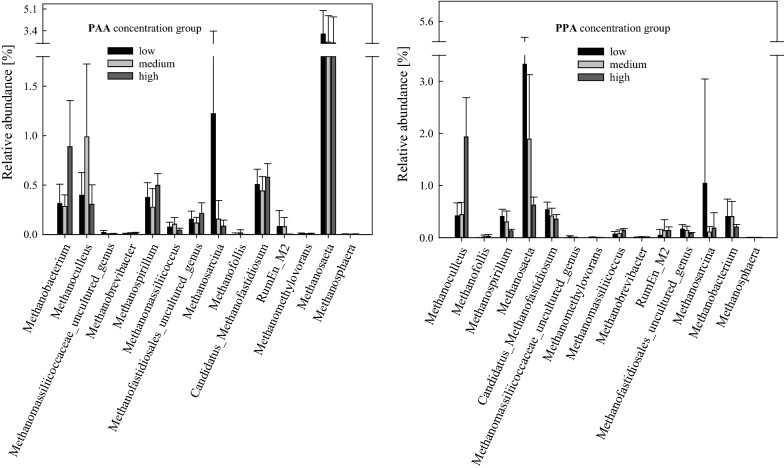
Relative sequence abundances [%] of mesophilic methanogens of the low, medium, and high PAA (left) and PPA (right) concentration groups. Bars represent mean values, whiskers standard deviations

For a detailed presentation and discussion of the gas properties of mesophilic samples, please refer to Wagner et al. [15]. Methane production was detected in all mesophilic samples. Complex protein samples under medium load conditions showed the highest cumulative methane production. Methane production was considerably restricted in medium-load amino acid samples and in high-load complex protein samples. 14 genera belonging to the phylum *Euryarchaeota* could be found in mesophilic samples. The mean relative abundance of this phylum ranged from 1.73 ± 0.27% in high-load ME samples on day 14 to 10.8 ± 1.03% in medium-load ME samples on day 28. The most dominant methanogenic genera were *Methanosarcina* spp. and *Methanosaeta* spp. (Fig. 2 and Additional file 1: Fig. S4). The genus *Methanosarcina* was predominant in samples fed with complex proteins under medium load conditions at the end of the incubation period, with a mean relative abundance of 5.61 ± 0.52% in Cas and 7.22 ± 1.55% in ME samples. By contrast, a relatively high abundance of hydrogenotrophic *Methanoculleus* spp. (and syntrophic bacterium *Tepidanaerobacter* spp.) could be observed in Cas samples under high load conditions (Additional file 1: Fig. S4). The mean sequence contribution of *Euryarchaeota* over all mesophilic sequences was 6.31 ± 2.47% in low, 4.98 ± 2.21% in medium, and 5.07 ± 2.11% in the high PAA concentration groups. On genus level, *Methanosarcina* spp. and *Methanosaeta* spp. were highest in low phenyl acid concentration groups (Fig. 2). The genera *Methanoculleus* and *Methanofollis* were positively (*p* < 0.05, B–H adjusted) correlated with PPA concentration (Fig. 1), and *Methanoculleus* spp. was a significant *LEfSe* biomarker for the high PPA concentration group as shown in Table 3.

### Thermophilic communities

#### Core microbiome and metagenomic biomarkers

A detailed description of the core microbiome of all substrates can be found in Table 1. Over all thermophilic samples, the genera *Defluviitoga* and DTU014 (*Clostridia*) were part of the core microbiome of each variant. The acetate-oxidising bacterium (SAOB) *Syntrophaceticus* spp. was part of the core microbiome of samples fed with amino acids (Tryp, Tyr, or Phe), whereas the SAOB *Tepidanaerobacter* spp. was part of the core microbiome of samples fed with complex proteins. By contrast, no SAOB could be found in the core microbiome of the controls (Table 1).

The metagenomic biomarkers (*LEfSe* analysis, *p* < 0.05) of all substrate variations are listed in Table 2. All the biomarkers calculated for samples fed with complex proteins were also part of the core microbiome of the respective samples (Tables 1 and 2).

#### Phenyl acids and community dynamics

Results of thermophilic phenyl acid formation were published previously [15] and are presented in a summarised form in Additional file 1: Fig. S6.

**Fig. 3:**
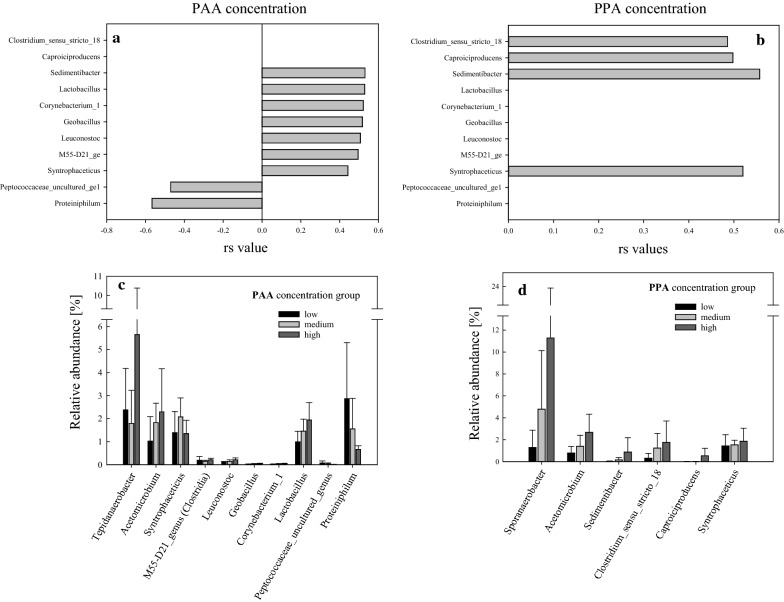
*Spearman*’s rank correlation coefficients (rs) including B-H adjustments between thermophilic genera and PAA (**a**) and between thermophilic genera and PPA (**b**) concentrations of day 28. OTUs with a standard deviation < 3 calculated over all samples were excluded. Relative sequence abundances of relevant *LEfSe* and *Spearman* genera of low, medium, and high PAA (**c**) and PPA (**d**) concentration groups. Bars represent mean values, whiskers the respective standard deviations

Compared with the mesophilic approach, considerably fewer microorganisms correlated with PAA and PPA concentrations. Except for *Clostridium*_sensu_stricto_18, the genera significantly correlating with phenyl acids during thermophilic incubation were different from those found during mesophilic incubation. PAA concentration positively correlated (*p* < 0.05) with the genera *Sedimentibacter*, *Lactobacillus*, *Leuconostoc*, M55-D21_genus, *Syntrophaceticus, Geobacillus,* and *Corynebacterium*_1 (Fig. 3). Additional information on the latter two genera can be looked up in Additional file 1: Text S3. PAA concentration negatively correlated (*p* < 0.05) with the genera *Peptococcaceae* (uncultured genus) and *Proteiniphilum*. The genera *Sedimentibacter* and *Syntrophaceticus* positively correlated with both PAA and PPA concentration. Moreover, Clostridium_sensu_stricto_18 and *Caproiciproducens* spp. positively correlated with PPA but not with PAA concentration. No negative (*p* < 0.05) correlations could be found between PPA concentration and thermophilic genera on day 28.

The genera *Sedimentibacter*, *Tepidanaerobacter*, *Acetomicrobium,* and *Sporanaerobacter* were significant *LEfSe* biomarkers (LDA ≥ 4) for mesophilic as well as thermophilic high phenyl acid concentration groups (Table 3). Moreover, *Lactobacillus* spp., DTU014_genus, and *Keratinibaculum* spp. were significant biomarkers for high PAA concentration group. From all the *LEfSe* biomarkers for high PAA concentration, *Tepidanaerobacter* spp. showed the highest abundance in the high PAA concentration group: the mean relative abundance ranged from 2.38 ± 1.80% in the low to 5.65 ± 4.74% in the high PAA concentration group (Fig. 3c). From all the *LEfSe* biomarkers for high PPA concentration, *Sporanaerobacter* spp. showed the highest mean relative abundance, ranging from 1.28 ± 1.59% in the low to 11.3 ± 12.5% in the high PPA concentration group (Fig. 3d). For information on thermophilic *LEfSe* biomarkers of the low and medium PAA and PPA concentration groups, please refer to Additional file 1: Table S1.

#### Methanogenic properties

**Fig. 4:**
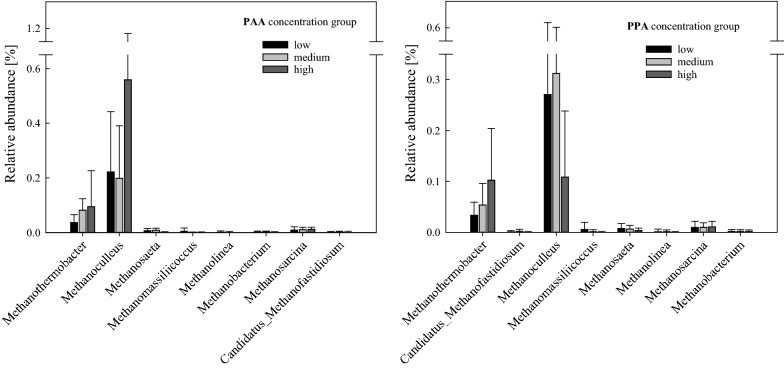
Relative sequence abundances [%] of thermophilic methanogens of the low, medium, and high PAA (left) and PPA (right) concentration groups. Bars represent mean values, whiskers standard deviations

For a detailed presentation and discussion of the gas properties of all thermophilic samples, please refer to Wagner et al. [15]. Methane production was observed in all thermophilic samples. The highest cumulative methane production was achieved in ME and Cas samples under high overload conditions, whereas the lowest cumulative methane yields could be observed in reactors fed with amino acids under medium overload conditions [15]. When looking at *Archaea* specifically, eight genera could be assigned to the phylum *Euryarchaeota* as shown in Fig. 4. The sequences of this phylum contributed with 0.28 ± 0.23% to the low, with 0.30 ± 0.18% to the medium, and with 0.67 ± 0.53% to the high PAA concentration group and with 0.33 ± 0.34% in the low, with 0.39 ± 0.29% in the medium, and with 0.23 ± 0.11% in the high PPA concentration group. The genera *Methanosarcina* spp. and *Methanothermobacter* spp. were the most abundant methanogens in thermophilic controls at day 0 with a sequence contribution of 0.04 ± 0.02% and 0.02%, respectively. Over the course of the incubation, *Methanoculleus* spp. became the most abundant methanogen over all thermophilic samples, followed by *Methanothermobacter* spp.; the highest abundances of these two genera were shown in the high PAA concentration group (Fig. 4). The relative abundance of *Methanosaeta* spp., which was very low in general, was even lower at elevated PAA concentrations.

### Prediction of metagenomic properties (piphillin)

The analysis inferred 250 OTUs which exceeded the identity threshold of 97%. Furthermore, 288 genomes and 359 KEGG pathways were observed for this data set, including all mesophilic as well as all thermophilic normalised samples. These numbers are comparable to a previous study focusing on the two meso- and thermophilic methanogenic systems [40].

**Fig. 5:**
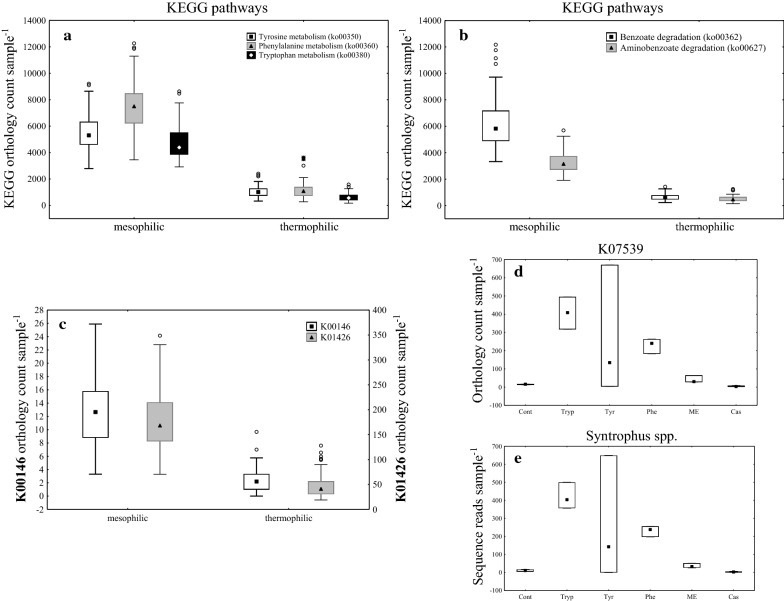
KEGG orthology counts of amino acid metabolism pathways (**a**), of (amino-) benzoate degradation pathways (**b**), and of the enzymes phenylacetaldehyde dehydrogenase (K00146) and amidase (K01426) (**c**) for all meso- and thermophilic samples. KEGG orthology counts of the enzyme 6-oxocyclohex-1-ene-carbonyl-CoA hydrolase (K07539) of mesophilic samples at low overload conditions on day 28 (**d**). Sequence abundance of the genus *Syntrophus* of mesophilic samples at low overload conditions on day 28 (**e**). The markers represent the median, the boxes show the upper–lower quartiles of each median, the whiskers the non-outlier range (coefficient 1), circles represent outliers, and stars extreme values

Generally, the orthology counts for peripheral and central benzoyl-CoA pathways were considerably higher in mesophilic than in thermophilic samples (Fig. 5a, b). The enzyme 6-oxocyclohex-1-ene-1-carbonyl-CoA hydrolase, responsible for the anaerobic benzene ring cleavage during benzoate degradation (KEGG orthology K07539) could only be found in mesophilic samples. More specifically, the enzyme was abundant (> 60 orthology counts sample^−1^) in Tryp, Tyr, Phe, and ME samples under low overload conditions at day 28, whereas all other mesophilic variants showed a low abundance. When present in high abundance (Fig. 5d), the orthologue could be assigned to the *Syntrophus acidotrophicus* genome (Fig. 5e).

The enzyme amidase (K01426), part of the PAA metabolism (Ko00360) and responsible for converting 2-phenylacetamide to PAA, was about tenfold more abundant than the enzyme phenylacetaldehyde dehydrogenase (K00146), which takes part in forming PAA out of phenylacetaldehyde (Fig. 5c). In mesophilic samples, K01426 could be assigned to the genome *Clostridium saccharolyticum* WM1 in Tyr samples under medium overload conditions for both day 14 and 28. The enzyme was also occasionally abundant in Cont, Phe, Cas, and ME samples and could be assigned to the genomes *Bradyrhizobium* sp. BF49 or *Petrimonas* sp. IBARAKI (Fig. 5c). In thermophilic samples, the enzyme K01426 was highly abundant in one Phe sample under medium overload conditions at day 28 and could be assigned to *Lactobacillus fermentum* IFO 3956.

## Discussion

### Mesophilic communities

The microbial diversity was relatively high in mesophilic samples (Additional file 1: Fig. S3). This is in accordance with previous studies, which showed that mesophilic communities tended to be more diverse and were thus considered less susceptible to disturbances [41, 42]. For further discussions according the microbial diversity of meso- and thermophilic samples, please refer to Additional file 1: Text S1.

Several microorganisms positively correlated with PAA and PPA concentrations (Fig. 1). For instance, the relative abundance of Candidatus *Caldatribacterium* (about 3%) and the PAA concentration [15] were highest in medium load Phe samples, which indicates that this microorganism was directly or at least indirectly involved in the conversion of phenylalanine to phenylacetate. This genus belongs to the phylum *Atribacteria* which is associated with sugar fermentation [43]. Ca. *Caldatribacterium* was also hypothesised to be acidogenic in thermophilic fermenters fed with Maotai-flavoured distillers’ grain, which is characterised by a low C/N ratio and a high organic matter content [44].

Phenyl acid degradation was more frequently observed in mesophilic than in thermophilic samples [15]. This is in accordance with *Piphillin* results, which indicated that the ring cleavage predominantly took place in mesophilic samples (Fig. 5). Ring cleavage could be associated with *Syntrophus acidotrophicus* (Fig. 5e), a genus that was also a significant biomarker of the low PPA concentration group (LDA = 3) and negatively correlated (*p* < 0.05) with PAA concentrations (rs = − 0.46). This indicates its significance for anaerobic benzene ring cleavage. The presence of the genus *Syntrophus* as well as the enzyme 6-oxocyclohex-1-ene-1-carbonyl-CoA hydrolase were not only restricted to mesophilic but also to low overload samples—irrespective of the substrate used (Additional file 1: Fig. S9). This indicates that high substrate loads can not only lead to higher phenyl acid concentrations in methanogenic systems, but also to a restricted benzene ring cleavage rate. However, this remains to be studied in more detail.

In mesophilic high overload samples, the relative abundances of hydrogenotrophic methanogens and SAOBs were relatively high. This indicates a switch towards SAO-induced hydrogenotrophic methanogenesis; however, a higher utilisation of acetate could not be observed and the methane production was relatively low in these samples [15]. The restricted acetoclastic performance is in accordance with previous studies, which showed that *Methanosarcina* spp. and especially *Methanosaeta* spp. are sensitive to typical overload indicators like high ammonium concentrations [8, 45]. The dominance of *Methanosarcina* spp. in samples fed with complex proteins at medium overload conditions can be explained by the fact that ammonium concentrations were still relatively low (about 2 g NH_4_-N L^−1^), while the acetate concentrations were sufficient (> 1 mM) during the first 14 days of incubation [8, 15]. *Methanosaeta* spp. was prevailing especially in low overload samples that were characterised by relatively low acetate and ammonium concentrations [15]. Interestingly, the acetate concentrations were still quite high (about 25 mM on day 0) for *Methanosaeta* spp. to be the dominant acetoclastic methanogen. This indicates that also other biochemical and microbial factors might influence the competitiveness of *Methanosaeta* spp. Results regarding direct negative effects of phenyl acids on methanogenic *Archaea* were inconclusive for mesophilic samples. It seems plausible that phenyl acids do not negatively affect all methanogens, but only some representatives of this group. The both negative and positive effects of phenyl acids on methanogens could also be linked to substrate overload conditions. However, this remains to be studied in more detail.

### Thermophilic communities

The microbial diversity in thermophilic samples was considerably lower than in mesophilic samples (Additional file 1: Fig. S3). The dominance (and also importance) of the genus *Defluviitoga* (phylum *Thermotogae*), which degrades carbohydrates to H_2_/CO_2_ and acetate, could be confirmed for thermophilic digesters [46–48].

*LEfSe* analyses showed that *Sedimentibacter* spp., *Tepidanaerobacter* spp., *Acetomicrobium* spp., and *Sporanaerobacter* spp. were significant biomarkers for both meso- as well as thermophilic phenyl acid formation (Table 3). The *LEfSe* algorithm is a useful and quite robust three-step tool to analyse metagenomics biomarkers. In this study, it not only elucidated which genera significantly differed between the classes (*Kruskal*–*Wallis* H-test), but also considered consistency (*Wilcoxon t* test) and biological relevance (LDA) [49]. LDA scores of 4 or higher were chosen to highlight the most relevant genera for describing the differences between the classes (and sub-classes). *Acetomicrobium hydrogeniformans* and *A. mobile* are anaerobic thermophiles known for their ability to degrade Phe; this can lead to an increase in PAA concentration [50–52]. When looking at the organism-specific pathways (Ko00360) in phenylalanine samples, *Acetomicrobium* (*mobile*) also contains the enzyme 2-enoate reductase (K10797) responsible for the transformation of trans-cinnamate to PPA. *Sporanaerobacter* spp. was previously isolated from a pit fermenting strong aromatic liquors at mesophilic temperatures [53]. *Tepidanaerobacter* spp. oxidises acetate in syntrophic association with a hydrogenotrophic methanogenic partner [54]; in the present study, SAO-induced hydrogenotrophic methanogenesis was the most important mineralising process in thermophilic reactors. In the present investigation, the substrate determined whether *Syntrophaceticus* spp. or *Tepidanaerobacter* spp. was the dominant SAOB. While *Syntrophaceticus* spp. was found in Tryp, Tyr, and Phe samples (Additional file 1: Fig. S7), *Tepidanaerobacter* spp. was found in Cas and ME samples (Table 1, Additional file 1: Fig. S8). The KEGG pathway ko00360 (phenylalanine metabolism) showed that *Tepidanaerobacter acetatoxydans* was potentially able to degrade 2-phenylacetamide to PAA via an amidase (K01426) [55]. In the present study, thermophilic *SAOBs* were identified as important players during the degradation of aromatic compounds; however, it remains to be elucidated whether they directly or indirectly contribute to the anaerobic phenyl acid turnover. Further discussion regarding thermophilic SAOBs can be found in Additional file 1: Text S2.

*Sedimentibacter* spp. further significantly correlated with high PAA concentrations (Fig. 3). *Spearman* correlation analyses showed that genera like *Leuconostoc* and *Lactobacillus*, next to *Sedimentibacter* spp. and *Syntrophaceticus* spp., were positively correlated with phenyl acid formation (Fig. 3). *Leuconostoc* spp. and *Lactobacillus* spp. belong to the order *Lactobacillales* and are described as (facultative) anaerobic lactic acid bacteria (LAB) [56–59]. These two genera are normally used as starter organisms in the production of fermented food. They are capable of producing PLA and PAA out of Phe and PLA out of Tyr [58, 60]. This was primarily described at temperatures around 30 °C. However, also thermophilic LAB exist that convert phenylalanine to PAA during cheese production [60]. Over all thermophilic samples, *piphillin* analyses showed that a catalase-peroxidase (K03782), responsible for the formation of 2-Phenylacetamide (out of Phe), and an amidase (K01426), responsible for the formation of PAA (out of 2-Phenylacetamide), were more abundant than the enzyme phenylacetaldehyde dehydrogenase (K00146). This indicates that—at least—two different strategies to anaerobically degrade Phe to PAA were possible in thermophilic samples.

*Sedimentibacter* spp. was not only shown to be involved in phenyl acid formation, but also in anaerobic (amino acid) degradation in meso- and thermophilic systems [61–66]. *S.* *hydroxybenzoicum*, isolated from freshwater sediments, was capable of anaerobically degrading phenolic compounds at mesophilic temperatures [65]. The results of this study confirmed that *Sedimentibacter* spp. is important in the dynamics of aromatic compound formation/degradation during meso- and thermophilic AD. *Proteiniphilum* spp. and *Peptococcaceae* (uncultured genus), which negatively correlated with PAA over all thermophilic samples on day 28 (Fig. 3), belong to the phyla *Bacteroidetes* and *Firmicutes*, respectively. Even though the family *Peptococcaceae* is associated with anaerobic benzene degradation [20], the breakdown of PAA and PPA by these microorganisms can be ruled out as their relative abundances were low at high phenyl acid concentrations. The relative abundances of *Proteiniphilum* spp. were also low at high PAA concentrations (Fig. 3c).

No negative correlations could be found between methanogens and phenyl acid concentrations in thermophilic samples. Thus, these results indicate that methanogens of the thermophilic approach were not impaired by the formed phenyl acids, which is in accordance with the biochemical data previously assessed [15]. When synoptically looking at both meso- and thermophilic reactors, the results implied that the ability of anaerobic phenyl acid formation is not restricted to a certain phylogenetic group of microorganisms but rather wider distributed in the domain *Bacteria*.

## Conclusions

For both meso- and thermophilic reactors, *Sedimentibacter* spp., *Tepidanaerobacter* spp., *Acetomicrobium* spp., and *Sporanaerobacter* spp. were shown to be significant biomarkers for high phenyl acid concentrations and thus considered to be involved in the degradation of amino acid and protein-rich precursor substrates. Members of the genus *Syntrophus* probably took part in the anaerobic benzene ring cleavage in mesophilic samples at low overload conditions (Additional file 1: Fig. S9). They might be important players in preventing phenyl acid accumulation and reactor performance deterioration. Acetoclastic methanogenesis dominated over all mesophilic samples. A shift from acetoclastic to SAO-induced hydrogenotrophic methanogenesis took place in thermophilic samples. This methanogenic pathway seemed to be the quite robust when proteinaceous materials/precursors were degraded in high loads. Interactions between microbes involved in the formation/degradation dynamics of aromatic compounds were highly complex. Further studies on phenyl acid formation dynamics are thus pending, especially when considering the influence of further factors like temperature, substrate, and substrate load. In further consequence, this knowledge would help to increase the energy exploitation of protein-rich (and lignocellulosic) wastes thus would contribute to a carbon-neutral, economically sustainable, and ethically acceptable energy management.

## Methods

### Experimental setup and sampling

The samples used in this study derived from an earlier work focusing on the formation of phenyl acids under mesophilic and thermophilic AD conditions [15]. In brief, batch bioreactors contained either Phe, Tyr, Tryp, ME, or Cas as additional substrate. The complex protein substrates ME and Cas were analysed in three (5.0, 20.0 and 50.0 g L^−1^) and the amino acids Phe, Tyr, and Tryp in two (1.0 and 10.0 g L^−1^) different final concentrations. According to the respective substrate load, the samples were grouped into low, medium, and high overload reactors [15]. A control was included containing no additional substrate. Experiments were carried out in triplicates. Samples were incubated at 52 °C or at 37 °C for 28 days. Further information on the experimental setup, inocula, lab-use substrates, methane yields, phenyl acid concentrations, and general biochemical properties can be found in the preceding work [15].

Considering the use of two inocula (thus two temperature regimes), three time measuring points, and various substrates at different load conditions [15], 234 samples in total were used for molecular analyses.

### DNA extraction

For molecular biological analyses, 1 mL samples were taken from each flask after 0, 14, and 28 days. The samples were stored at − 20 °C until extraction. After thawing, the samples were centrifuged at 20,000×*g* for 15 min. Each pellet was washed in 900 µL sterile phosphate buffer (1×) solution (per litre: 8 g NaCl, 0.2 g KCl, 1.4 g Na_2_HPO_4_, 0.2 g KH_2_PO_4_, pH 7.4), transferred into bead tubes (Macherey–Nagel, Germany) and centrifuged again at 11,000×*g* for 10 min. The phosphate buffer was discarded, and DNA extraction was conducted according to the manufacturer’s instructions of the Soil Extract II Kit (Macherey–Nagel, Germany). The lysis buffer SL-1 (700 µL) and the enhancer (50 µL) were added to the washed pellet. Cell lysis took place in a FastPrep-24™ 5G (MP Biomedicals, USA) for 1 × 30 s (5 m s^−1^). The DNA was eluted in 50 µL elution buffer. DNA quantity and quality were measured via NanoDrop 2000c™ (ThermoFisher Scientific, USA) system. The DNA extracts were diluted to reach a working concentration of 2.5 ng µL^−1^.

### NGS library and sequencing

A simple DNA profiling approach [67, 68] was conducted with all variants of day 0 in order to check for the same microbial community structure at the beginning of the experiment. Controls of day 0 and all samples of day 14 and 28 were used for *next*-*generation* sequencing (NGS) analyses. The NGS library preparation was conducted in-house. The small subunit rRNA gene primers 515f and 806r [69], according the Earth microbiome project [70], were used to target the V4 region. The first PCR step, including the 16S rRNA primers and the Illumina^®^ adapter sequences, was performed as described previously [40]. 25 µL PCR solution contained 12 µL PCR Mix (MyTaq™ Mix 2× (Bioline), 250 nM of each primer–adapter combination, 20% Betaine Enhancer Solution (5×) (VWR International, Germany), and PCR-grade water to reach a final volume of 24 µL, as well as 1 µL DNA template (2.5 ng DNA µL^−1^). The quality of the PCR products was checked with a 1.5% agarose gel. The PCR products of the first step were diluted 1:5 and used as template for a second amplification. For that purpose, the Illumina^®^ barcodes (i5 and i7) were attached. The same PCR procedure as in the first PCR step [40] was conducted except that only five cycles were applied and that the annealing temperature was set to 56 °C. PCR products were again checked with a 1.5% agarose gel. Subsequently, final PCR products were quantified fluorometrically as described previously [71].

PCR products of each sample (15 ng) were pooled, purified with Hi Yield^®^ Gel/PCR DNA Fragment Extraction Kit (SLG^®^, Germany), and eluted in 50 µL Tris–HCl buffer. The DNA quantity was again measured via QuantiFluor^®^ dsDNA Dye (Promega, Germany). Co-extraction of contaminants was checked via the NanoDrop 2000c™ system. The final ready-to-load sample pool showed a DNA concentration of 14 ng µL^−1^ (260/280 absorbance ratio: 1.88) and was subsequently sent to Microsynth AG in Switzerland where the sequencing was done according to the company’s protocols.

### Reads procession and OTU classification

Raw sample reads were processed using the program mothur [72] (v.1.39.5 as well as v.1.42.1 for pre-clustering and chimera search) and the MiSeq SOP (March 2019) [73]. A contig file was created with the paired ends (10,672,059 sequences in total, 65,877 ± 12,374 sequences sample^−1^). After quality filtering (approx. 15% of the sequences were discarded), unique sequences were aligned to the SILVA V132 database [74]. After another quality check and pre-clustering [75], chimeric amplicons were removed applying the *vsearch* algorithm (VSEARCH v2.13.3.) [76]. Sequence classification was done with the *k*-*nearest neighbor* (knn) algorithm. Sequences were clustered to OTUs based on their taxonomy. For a better comparability of samples while simultaneously ensuring an adequate coverage of the species richness, rarefaction curves were checked, and samples were normalised to 19,351 reads sample^−1^. Two samples, both deriving from the thermophilic community on day 14, were excluded from further analyses due to an insufficient sequencing depth (*n* < 3060 sequences per sample). The *Mantel* test (Gower similarity index) showed that the communities prior to and after rarefaction did not differ significantly (*R* = 0.94, *p* < 0.01, *N* = 9999).

### Mock communities

Three different, defined MOCK communities were included to validate the NGS procedure. The ZymoBIOMICS™ Microbial Community standard (Zymo, containing eight bacterial and two yeast microorganisms, further referred to as Mock1) and the archaeon *Methanosarcina thermophila* DSM 1825 (DSMZ, German Collection of Microorganisms and Cell Cultures, further referred to as Mock2) were analysed separately as well as in combination (50% genomic DNA Zymo, 50% genomic DNA *M. thermophila,* further referred to as Mock3).

The MOCK communities were co-processed with reactor samples. All bacterial and archaeal microorganisms of the three MOCK communities (Mock1, Mock2 and Mock3) could be recovered at genus level. Therefore, the validity and reliability of the applied strategies for DNA extraction, library preparation, and data processing were proven.

### Prediction of metagenomic properties

After subsampling to 19,351 reads per sample, a sequence file containing only representative sequences and an OTU abundance table were generated via mothur (version 1.42.1.). The tool *piphillin* (https://piphillin.secondgenome.com, July 2019), which uses the nearest-neighbor algorithm to pair 16S rRNA gene sequences with genomes [77] was applied. The analyses focused on metagenomic predictions of the peripheral (KEGG orthology ko00350, ko00360, and ko00380) and central (KEGG orthology ko00362 and ko00627) benzoyl-CoA pathways. Moreover, metagenomics prediction of the enzyme 6-oxocyclohex-1-ene-1-carbonyl-CoA hydrolase (KEGG orthology K07539), associated with anaerobic benzene ring cleavage, was also included. The program used USEARCH v8.1.1861 [28] and an identity cut-off of 97%. The KEGG database (version October, 2018) was used as a Ref. [55].

### Graphical and statistical analyses

The Mock community (*n* = 9) check as well as the ecological diversity analyses (Shannon–Weaver index) were done with *RStudio*^*®*^ using the packages *ggplot2* and *phyloseq* [78].

Thenceforth, meso- and thermophilic data were analysed separately; only OTU’s with a total abundance of ≥ 10 were used for each temperature regime (abundance sample^−1^ of removed OTUs: ≤ 5). In mothur, the *LEfSe* [49] and *get.coremicrobiome* command were used to further analyse communities on a metagenomic basis. For the general description of biomarkers via *LEfSe*, the substrate was set as class and the degree of overload (low, medium, high) as subclass. Biomarker discovery for samples with low, medium, and high phenyl acid production was done via *k*-*means* clustering of PAA and PPA concentrations (low: 0–2.66, medium: 2.74–9.35, and high: 11.9–23.2 mM PAA; low: 0–1.98, medium: 2.07–6.97, and high: 7.56–21.4 mM PPA). The *LEfSe* algorithm uses the *Kruskal*–*Wallis H*-test [79] for detecting biomarkers for the respective class. Within each class, the pairwise *Wilcoxon t*-test [80] is used for detecting biomarkers of subclasses. The *LEfSe* algorithm also includes linear discriminant analyses (LDA) to further estimate the magnitude of each effect (thus also takes the effect size into consideration) [49].

*Spearman* correlation analyses (done for samples of day 28), *k*-*means* clustering, and the *Mantel* test were done with *PAST*^®^ 3 [81]. For *Spearman*’s rank correlations coefficient analyses, OTUs showing low variation (standard deviation < 3), calculated over all samples of the respective temperature regime, were excluded to reduce background noise. For correlation analyses, the B–H procedure [82] was applied in Microsoft^®^ Excel^®^. For *Spearman*’s rank correlations, the biochemical and OTU data were log (*x* + 1) and Box–Cox (*x* + 1) transformed, respectively.

Significant microorganisms for samples with low and high phenyl acid production were processed with the program STAMP 2.1.3 [83]. For that purpose, the White’s non-parametric *t*-test (two-sided) was used [84]. When considering effect sizes, genera with a proportion difference below 1 were excluded. Confidence intervals were provided via percentile bootstrapping (1000 permutation test replicates). The false discovery rate was controlled with the B–H adjustment.

Graphical presentations of phenyl acid formation and of *piphillin* analyses were done with *Statistica™ 13* (TIBCO^®^ Software Inc.). All other figures were prepared with *SigmaPlot™ 14* (Systat^®^ Software Inc). The KRONA tool was used for interactive visualisations of relative sequences abundances [85].

## Supplementary information

**Additional file 1: Table S1.** Significant *LEfSe* biomarker with a LDA score greater or equal 4 for low and medium PAA and PPA concentration groups. **Figure S1.** Mean sequence proportions [%] of significant mesophilic phyla of low and high PAA and PPA concentration groups. **Figure S2.** Mean sequence proportions [%] of significant thermophilic phyla of low and high PAA and PPA concentration groups. **Figure S3.** Shannon diversity index for Cont, Tryp, Tyr, Phe, ME, and Cas samples over all measuring time points under low, medium, and high overload conditions. **Figure S4.** Interactive visualisation of mesophilic taxa of the controls as well as of the Tryp, Tyr, Phe, ME, and Cas samples at low, medium, and high overload conditions on day 28. **Figure S5.** Interactive visualisation of thermophilic taxa of the controls as well as of the Tryp, Tyr, Phe, ME, and Cas samples at low, medium, and high overload conditions on day 28. **Figure S6.** Concentrations of PAA and PPA of mesophilic and thermophilic samples on day 0, 14, and 28. **Figure S7.** Relative sequence abundance [%] of *Syntrophaceticus* spp. in thermophilic low, medium, and high overload samples on day 28. **Figure S8.** Relative sequence abundance [%] of *Tepidanaerobacter* spp. in thermophilic low, medium, and high overload samples on day 28. **Figure S9.** Relative sequence abundance [%] of *Syntrophus* spp. in mesophilic low, medium, and high overload samples. **Text S1.** Differences in microbial diversity between meso- and thermophilic communities. **Text S2.** SAO- induced hydrogenotrophic methanogenesis in thermophilic samples. **Text S3.** Further positive *Spearman* correlations between phenyl acid formation and thermophilic genera.

## Data Availability

Mesophilic and thermophilic sequences were uploaded to GenBank^®^ via the submission tool BankIt (BioProject ID for mesophilic samples: 564060, BioProject ID for thermophilic samples: 564063).

## Abbreviations

SAO: Syntrophic acetate oxidation
Tryp: Tryptophan
Tyr: Tyrosine
Phe: Phenylalanine
PAA: Phenylacetate
PPA: Phenylpropionate
PLA: Phenyllactate
KEGG: Kyoto Encyclopedia of Genes and Genomes
AD: Anaerobic digestion
ME: Meat extract
Cas: Casein
OTU: Operational taxonomic unit
Cont: Control
LDA: Linear discriminant analysis
*LEfSe*: Linear discriminant analysis effect size
B–H: Benjamini–Hochberg
SAOB: Syntrophic acetate-oxidising bacterium
LAB: (Facultative) anaerobic lactic acid bacteria
Zymo: ZymoBIOMICS™ Microbial Community standard
DSMZ: German Collection of Microorganisms and Cell Cultures
rs: *Spearman*’s rank correlations coefficient
