# Supplementary material for: Microbial community dynamics in mesophilic and thermophilic batch reactors under methanogenic, phenyl acid-forming conditions
Source: Biotechnol Biofuels. 2020 May 6;13:81. doi: 10.1186/s13068-020-01721-z (PMC7201606; doi:10.1186/s13068-020-01721-z)
Supplement: Supplementary file 1 — Additional file 1: Table S1. Significant LEfSe biomarker with a LDA score greater or equal 4 for low and medium PAA and PPA concentration groups. Figure S1. Mean sequence proportions [%] of significant mesophilic phyla of low and high PAA and PPA concentration groups. Figure S2. Mean sequence proportions [%] of significant thermophilic phyla of low and high PAA and PPA concentration groups. Figure S3. Shannon diversity index for Cont, Tryp, Tyr, Phe, ME, and Cas samples over all measuring time points under low, medium, and high overload conditions. Figure S4. Interactive visualisation of mesophilic taxa of the controls as well as of the Tryp, Tyr, Phe, ME, and Cas samples at low, medium, and high overload conditions on day 28. Figure S5. Interactive visualisation of thermophilic taxa of the controls as well as of the Tryp, Tyr, Phe, ME, and Cas samples at low, medium, and high overload conditions on day 28. Figure S6. Concentrations of PAA and PPA of mesophilic and thermophilic samples on day 0, 14, and 28. Figure S7. Relative sequence abundance [%] of Syntrophaceticus spp. in thermophilic low, medium, and high overload samples on day 28. Figure S8. Relative sequence abundance [%] of Tepidanaerobacter spp. in thermophilic low, medium, and high overload samples on day 28. Figure S9. Relative sequence abundance [%] of Syntrophus spp. in mesophilic low, medium, and high overload samples. Text S1. Differences in microbial diversity between meso- and thermophilic communities. Text S2. SAO- induced hydrogenotrophic methanogenesis in thermophilic samples. Text S3. Further positive Spearman correlations between phenyl acid formation and thermophilic genera. [file 13068_2020_1721_MOESM1_ESM.docx]

Supplementary Material

Title: Microbial community dynamics in mesophilic and thermophilic batch reactors under methanogenic, phenyl acid- forming conditions.

Authors:

Eva Maria Prem^*1^, Blaz Stres^2,3,4^, Paul Illmer^1^, and Andreas Otto Wagner^1^

Affiliation:

^1^Department of Microbiology, Universität Innsbruck, Innsbruck, Austria

^2^Department of Animal Science, Biotechnical Faculty, University of Ljubljana, Jamnikarjeva 101, SI-1000 Ljubljana, Slovenia

^3^Institute of Sanitary Engineering, Faculty of Civil and Geodetic Engineering, University of Ljubljana, Jamova 2, SI-1000 Ljubljana, Slovenia

^4^Department of Automation, Biocybernetics and Robotics, Jozef Štefan Institute, Jamova 39, SI-1000 Ljubljana, Slovenia

^*^Corresponding author:

Eva Maria Prem, Department of Microbiology, Universität Innsbruck, Technikerstraße 25d, 6020 Innsbruck, Austria, Tel.: 0043 512 507 51346, email: [eva.prem@uibk.ac.at](mailto:eva.prem@uibk.ac.at)

Supplementary Results

|  | PAA | Sample size | Significant biomarkers  LDA ≥ 4 | PPA | Sample size | Significant biomarkers  LDA ≥ 4 |
| --- | --- | --- | --- | --- | --- | --- |
| mesophilic | Low | 51 | *Paludibacteraceae* (uncultured genus)  *Methanosarcina*  *Ruminiclostridium*  *Cloacimonadaceae*_W5 | Low | 61 | *Macellibacteroides*  *Paludibacteraceae* (uncultured genus)  Candidatus *Cloacimonas*  *Methanosaeta*  *Bacteroidetes*_vadinHA17_genus  *Ruminiclostridium*_1  *Cloacimonadaceae*_W5  *Synergistaceae* (uncultured genus)  *Proteiniphilum*  *Methanosarcina*  *Ruminiclostridium*  W27_genus (*Cloacimonadales*)  *Christensenellaceae*_R-7_group |
|  | Medium | 18 | *Anaerosalibacter*  *Terrisporobacter*  MBA03_genus (*Clostridia*)  *Clostridiales*_FamilyXI (uncultured_genus)  *Ruminococcaceae*_uncultured_genus  *Tepidimicrobium*  *Methanoculleus* | Medium | 15 | *Sedimentibacter*  *Bacteroides*  *Haloimpatiens*  *Tyzzerella*  *Clostridium*_sensu_stricto_15  *Crassaminicella*  *Tissierella*  *Streptococcus*  *Peptostreptococcus* |
| thermophilic | Low | 51 | *Caldicoprobacter*  *Clostridiales*_vadinBB60_group_genus  *Clostridium*_sensu_stricto_1  *Proteiniphilum*  MBA03_genus (*Clostridia*) | Low | 36 | *Clostridiales*_vadinBB60_group_genus |
|  | Medium | 19 | *Tepidimicrobium*  *Ruminiclostridium*  *Firmicutes* (uncultured genus)  *Syntrophaceticus*  *Sedimentibacter* | Medium | 28 | *Caldicoprobacter*  *Gelria*  *Ruminiclostridium* |

**Table S1**: Significant *LEfSe* biomarker with a LDA score ≥ 4 for low and medium PAA (left) and PPA (right) concentration groups.

**Figure S1** Mean sequence proportions [%] of significant mesophilic phyla of low and high PAA (upper row) and PPA (lower row) concentration groups. The 95% confidence intervals and p values were calculated via White’s non -parametric t-test (two-sided) including bootstrapping (95%) and a B-H adjustment. Samples with an effect size ≥1 were considered for visualisation.

**Figure S2** Mean sequence proportions [%] of significant thermophilic phyla of low and high PAA (upper row) and PPA (lower row) concentration groups. The 95% confidence intervals and p values were calculated via White’s non-parametric t-test (two-sided) including bootstrapping (95%) and a B-H adjustment.

**Figure S3** Shannon diversity index for Cont, Tryp, Tyr, Phe, ME, and Cas samples over all measuring time points under low (left), medium (middle), and high (right) overload conditions.

**Figure S4** Interactive visualisation of mesophilic taxa of the controls as well as of the Tryp, Tyr, Phe, ME, and Cas samples at low, medium, and high (ME, Cas) overload conditions on day 28.

**Figure S5** Interactive visualisation of thermophilic taxa of the controls as well as of the Tryp, Tyr, Phe, ME, and Cas samples at low, medium, and high (ME, Cas) overload conditions on day 28.

**Figure S6** Concentrations of PAA and PPA [1] of mesophilic (A) and thermophilic (B) samples on day 0, 14, and 28. Results are shown for Cont (low overload), for Tryp, Tyr, and Phe (medium overload), and for Cas and ME (high overload) samples. Middle points show medians, the boxes represent the lower - upper quartiles of each median.

**Figure S7** Relative sequence abundances [%] of *Syntrophaceticus* spp. in thermophilic low (left), medium (middle), and high (right) overload samples on day 28.

**Figure S8**: Relative sequence abundances [%] of *Tepidanaerobacter* spp. in thermophilic low (left), medium (middle), and high (right) overload samples on day 28.

**Figure S9**: Relative sequence abundances [%] of *Syntrophus* spp. in mesophilic low, medium, and high overload samples.

Figure S1

Figure S2


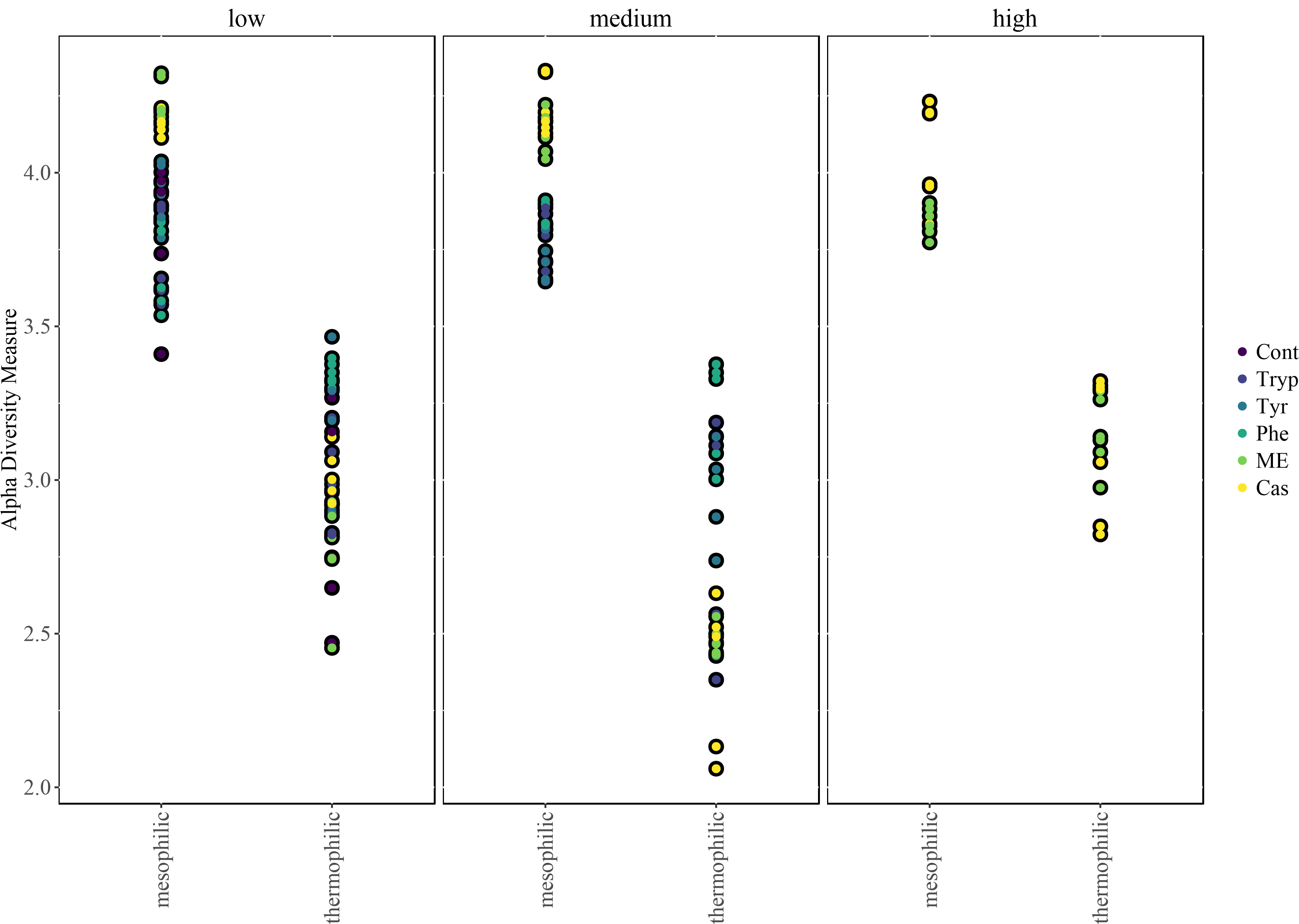
Figure S3

Figure S4

Figure S5

Figure S6

Figure S7

Figure S8

Figure S9


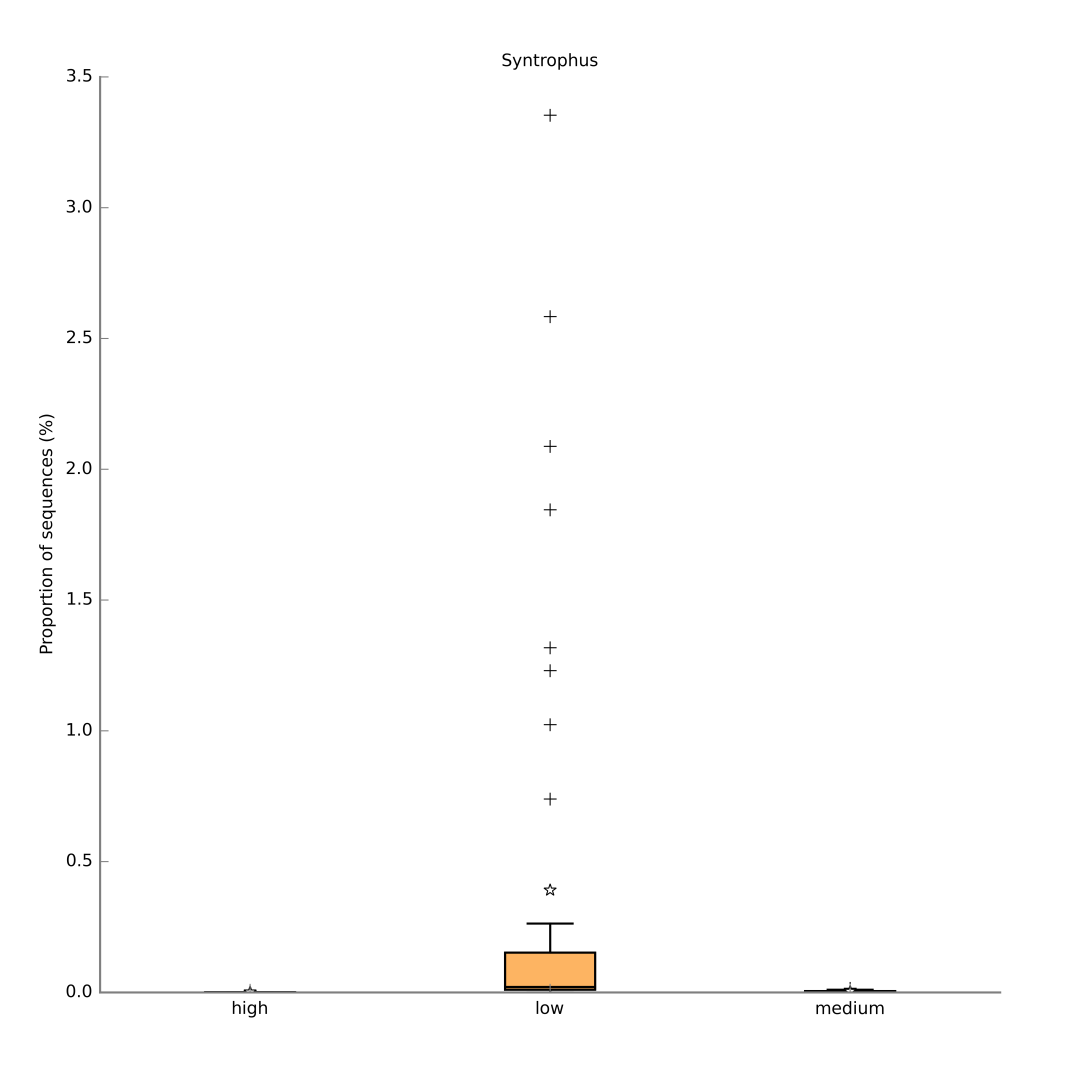


Supplementary Discussion

**Text S1:** Differences in microbial diversity between meso- and thermophilic communities

The microbial diversity in mesophilic samples was higher than that in thermophilic samples (Fig. S3). Previous studies showed that mesophilic communities are diverse and thus quite susceptible to disturbances [2, 3]. However, a high microbial diversity / functional redundancy could also indicate a circumvention / counteraction of suboptimal environmental conditions, meaning that highly effective species cannot dominate over other microorganisms [4, 5]. The mesophilic communities of this study derived from a wastewater treatment plant operated at low organic loading rates [6]. Suboptimal conditions were thus prevalent in most mesophilic samples fed with additional substrate; this consequently led to a higher microbial diversity (Fig. S3). By contrast, thermophilic (bacterial) communities are considered highly specialised and effective consortia [2, 7–9]. The relatively low diversity in thermophilic samples indicates that the incubation conditions were more favourable for thermo- than for mesophilic microorganisms and that few thermophilic taxa (like *Defluviitoga* spp.) were able to dominate the microbial consortium.

**Text S2:** Syntrophic acetate oxidation (SAO)-induced hydrogenotrophic methanogenesis in thermophilic samples

The anaerobic digestion of protein-rich substrates often leads to high free ammonia (NH_3_) and sulphide concentrations [10, 11]. Acetoclastic methanogens and especially *Methanosaeta* spp. are very sensitive towards high ammonia concentrations [11–14]. This often leads to a shift from acetoclastic to SAO- induced hydrogenotrophic methanogenesis [10, 11, 15] as also observed in thermophilic high load samples (Fig. S5). The hydrogenotrophic methanogen *Methanoculleus* spp. required an acetate-oxidising partner to make use of acetate. *Syntrophaceticus (schinkii)* and *Tepidanaerobacter (acetatoxydans)* were previously shown to oxidise acetate in syntrophic associations with hydrogenotrophic methanogens [16–18]. Interestingly, *Syntrophaceticus* spp. was only a core member in samples fed with amino acids (Tryp, Tyr, and Phe samples), whereas *Tepidanaerobacter* spp. was only a core member in microbiomes fed with complex proteins (Cas and ME samples) as shown in the main text (Table 1). Moreover, *Tepidanaerobacter* spp. was also a significant biomarker for Cas samples. At this stage, it is not clear which biochemical and/or microbial parameters caused these differences. Further studies are thus pending not only to elucidate the competitiveness of various SAOBs during syntrophic acetate oxidation, but also to understand their interactions / interdependencies with microorganisms engaged in preceding degradation phases and with methanogens.

**Text S3:** Further positive Spearman correlations between phenyl acid formation and thermophilic genera

The genus *Geobacillus* includes thermophilic and catabolically versatile species, which can be found in various sites like composts or hot springs [19]. It is plausible that *Geobacillus* spp. took part in the formation of PAA under anaerobic (batch) conditions (Fig. 2). However, *Geobacillus* spp. (and also *Corynebacterium*_1) were rare over all thermophilic samples (Fig. S5) and should thus be discussed tentatively at this stage. Analyses permitting discussions on species level, like shotgun sequencing, as well as pure culture approaches might be helpful to get more insight into their role in PAA formation under anaerobic conditions.

References

1. Wagner AO, Prem EM, Markt R, Kaufmann R, Illmer P. Formation of phenylacetic acid and phenylpropionic acid under different overload conditions during mesophilic and thermophilic anaerobic digestion. Biotechnol Biofuels. 2019;12:359. doi:10.1186/s13068-019-1370-6.

2. Sekiguchi Y, Kamagata Y, Syutsubo K, Ohashi A, Harada H, Nakamura K. Phylogenetic diversity of mesophilic and thermophilic granular sludges determined by 16S rRNA gene analysis. Microbiology (Reading, Engl ). 1998;144 (Pt 9):2655–65. doi:10.1099/00221287-144-9-2655.

3. Labatut RA, Angenent LT, Scott NR. Conventional mesophilic vs. thermophilic anaerobic digestion: A trade-off between performance and stability? Water Research. 2014;53:249–58. doi:10.1016/j.watres.2014.01.035.

4. Briones A, Raskin L. Diversity and dynamics of microbial communities in engineered environments and their implications for process stability. Chemical biotechnology ● Pharmaceutical biotechnology. 2003;14:270–6. doi:10.1016/S0958-1669(03)00065-X.

5. Vrieze J de, Christiaens MER, Walraedt D, Devooght A, Ijaz UZ, Boon N. Microbial community redundancy in anaerobic digestion drives process recovery after salinity exposure. Water Research. 2017;111:109–17. doi:10.1016/j.watres.2016.12.042.

6. Wagner AO, Markt R, Puempel T, Illmer P, Insam H, Ebner C. Sample preparation, preservation, and storage for volatile fatty acid quantification in biogas plants. Eng. Life Sci. 2017;17:132–9. doi:10.1002/elsc.201600095.

7. Moset V, Poulsen M, Wahid R, Højberg O, Møller HB. Mesophilic versus thermophilic anaerobic digestion of cattle manure: methane productivity and microbial ecology. Microb Biotechnol. 2015;8:787–800. doi:10.1111/1751-7915.12271.

8. d. Yu, Kurola JM, Lähde K, Kymäläinen M, Sinkkonen A, Romantschuk M. Biogas production and methanogenic archaeal community in mesophilic and thermophilic anaerobic co-digestion processes. Journal of Environmental Management. 2014;143:54–60. doi:10.1016/j.jenvman.2014.04.025.

9. Fernández-Rodríguez J, Pérez M, Romero LI. Comparison of mesophilic and thermophilic dry anaerobic digestion of OFMSW: Kinetic analysis. Chemical Engineering Journal. 2013;232:59–64. doi:10.1016/j.cej.2013.07.066.

10. Fotidis IA, Karakashev D, Angelidaki I. The dominant acetate degradation pathway/methanogenic composition in full-scale anaerobic digesters operating under different ammonia levels. International Journal of Environmental Science and Technology. 2014;11:2087–94. doi:10.1007/s13762-013-0407-9.

11. Westerholm M, Moestedt J, Schnürer A. Biogas production through syntrophic acetate oxidation and deliberate operating strategies for improved digester performance. Applied Energy. 2016;179:124–35. doi:10.1016/j.apenergy.2016.06.061.

12. Karakashev D, Batstone DJ, Trably E, Angelidaki I. Acetate oxidation is the dominant methanogenic pathway from acetate in the absence of Methanosaetaceae. Applied and Environmental Microbiology. 2006;72:5138–41. doi:10.1128/AEM.00489-06.

13. Karakashev D, Batstone DJ, Angelidaki I. Influence of environmental conditions on methanogenic compositions in anaerobic biogas reactors. Applied and Environmental Microbiology. 2005;71:331–8. doi:10.1128/AEM.71.1.331-338.2005.

14. Rajagopal R, Massé DI, Singh G. A critical review on inhibition of anaerobic digestion process by excess ammonia. Bioresour Technol. 2013;143:632–41. doi:10.1016/j.biortech.2013.06.030.

15. Moestedt J, Påledal S, Schnürer A, Nordell E. Biogas Production from Thin Stillage on an Industrial Scale—Experience and Optimisation. Energies. 2013;6:5642–55. doi:10.3390/en6115642.

16. Westerholm M, Roos S, Schnürer A. Tepidanaerobacter acetatoxydans sp. nov., an anaerobic, syntrophic acetate-oxidizing bacterium isolated from two ammonium-enriched mesophilic methanogenic processes. Syst Appl Microbiol. 2011;34:260–6. doi:10.1016/j.syapm.2010.11.018.

17. Westerholm M, Roos S, Schnürer A. Syntrophaceticus schinkii gen. nov., sp. nov., an anaerobic, syntrophic acetate-oxidizing bacterium isolated from a mesophilic anaerobic filter. FEMS Microbiol.Lett. 2010;309:100–4. doi:10.1111/j.1574-6968.2010.02023.x.

18. Westerholm M, Isaksson S, Karlsson Lindsjö O, Schnürer A. Microbial community adaptability to altered temperature conditions determines the potential for process optimisation in biogas production. Applied Energy. 2018;226:838–48. doi:10.1016/j.apenergy.2018.06.045.

19. Hussein AH, Lisowska BK, Leak DJ. Chapter One - The Genus Geobacillus and Their Biotechnological Potential. In: Sariaslani S, Gadd GM, editors. Advances in Applied Microbiology: Academic Press; 2015. p. 1–48. doi:10.1016/bs.aambs.2015.03.001.
